# Comparison of PCR‐Based Methods for the Detection of Canned Tuna Species

**DOI:** 10.1111/1750-3841.70424

**Published:** 2025-09-19

**Authors:** Chloe P. Castanon, Denise Hernandez, Akshay N. Khetrapal, Rosalee S. Hellberg

**Affiliations:** ^1^ Food Science Program, Schmid College of Science and Technology Chapman University Orange California USA

## Abstract

Canned tuna is susceptible to mislabeling due to its high consumer demand, complex global supply chains, and range of prices. DNA barcoding of a short fragment of the mitochondrial control region (CR), termed the CR mini‐barcode, has been established as an effective method for tuna species identification. However, the high level of DNA degradation in canned tuna products reduces the effectiveness of this method. Therefore, this study aimed to compare CR mini‐barcoding to targeted (i.e., real‐time or multiplex) PCR‐based methods to determine the most effective approach for canned tuna species identification. DNA was extracted in duplicate from 24 canned tuna products labeled as albacore, skipjack, yellowfin, and light tuna. Each sample was analyzed with CR mini‐barcoding, real‐time PCR, and multiplex PCR. The top‐performing targeted method also underwent sensitivity testing using binary species mixtures. Real‐time PCR showed the highest species identification rate, with 100% of products detected, followed by CR mini‐barcoding (33%) and multiplex PCR (29%). Real‐time PCR also showed excellent sensitivity, detecting 0.1%–1% of the target species in fresh and heat‐treated binary species mixtures. Multiplex PCR and real‐time PCR showed similar effectiveness in terms of cost and time, with a price of US$6 per sample and a total time of 3–6 h when testing all targeted species. Although CR mini‐barcoding required greater costs and time, it allowed for sequencing‐based detection of a range of species in the products. In conclusion, a combination of real‐time PCR and CR mini‐barcoding is recommended to allow for rapid screening of target species along with sequencing‐based confirmation.

**Practical Applications:**

This research provides a practical recommendation regarding the use of genetic methods for detecting species in canned tuna. Implementation of the recommended methodology is expected to enhance consumer protection and help regulatory agencies enforce accurate labeling.

## 1 Introduction

Tuna is a highly valued commodity, with a record 8.3 million tonnes of tuna and tuna‐like species captured globally in 2022 (FAO 2024). Whole and canned tuna are some of the top imported seafood products in the United States: close to 300,000 tonnes of tuna were imported in 2020, the majority (71%) of which was canned (NMFS 2022). Canned tuna is the third most consumed form of seafood in the United States (NFI 2024a), with the top canned tuna products by volume being light tuna and albacore (white) tuna (*Thunnus alalunga*) (NFI 2024b; NMFS 2022). Canned light tuna is mainly composed of skipjack tuna (*Katsuwonus pelamis*) but may also consist of other tuna species, such as yellowfin (*Thunnus albacares*), bigeye (*Thunnus obesus*), or longtail (*Thunnus tonggol*) (Bumble Bee Foods 2024; Chicken of the Sea 2016).

Canned tuna is susceptible to mislabeling due to factors such as high demand, complex global supply chains, and range of prices (Roungchun et al. 2022). Because the morphological features are removed during processing, canned tuna species are difficult to identify based on visual appearance alone (Chuang et al. 2012; Mitchell and Hellberg 2016). Canned tuna mislabeling may be intentionally carried out for economic gain, or it may be a result of the accidental inclusion of species harvested or processed in the same environment as the declared species (Roungchun et al. 2022). Previous research has reported mislabeling of canned tuna products globally, including species substitution and detection of undeclared species (Bojolly et al. 2017; Botti and Giuffra 2010; Chang et al. 2021; Emmi et al. 2023; Giusti et al. 2023; Klapper et al. 2023; Lee et al. 2022; Mariani et al. 2015; Mitchell and Hellberg 2016; Roungchun et al. 2022; Servusova and Piskata 2021; Sotelo et al. 2018; Wang et al. 2024).

Beyond the economic consequences, mislabeling canned tuna poses health risks, such as mercury exposure in vulnerable populations, and undermines conservation efforts. For example, pregnant women in the United States are advised to avoid bigeye tuna consumption and limit yellowfin, albacore, and canned white tuna to one serving per week due to potential mercury exposure (EPA–FDA 2024). Alternatively, these at‐risk individuals are encouraged to consume two to three servings per week of low‐mercury fish, such as canned light or skipjack tuna. Therefore, mislabeling higher mercury tuna as a lower mercury species can potentially result in elevated mercury exposure among at‐risk consumers (Burger and Gochfeld 2004; Emmi et al. 2023). Furthermore, mislabeling of tuna species may interfere with conservation efforts by enabling the sale of fish harvested from illegal, unreported, and unregulated (IUU) fishing, depleting vulnerable or endangered stocks of fish, and misrepresenting the abundance of the labeled species (Cawthorn et al. 2018; Cusa et al. 2021; Gordoa et al. 2017; Roungchun et al. 2022; Stawitz et al. 2017). For instance, previous research has reported the detection of the endangered southern bluefin tuna (*Thunnus maccoyii*) or near‐threatened Pacific bluefin tuna (*Thunnus orientalis*) mislabeled as yellowfin or ahi tuna (Kitch et al. 2023; Liou et al. 2020; Roungchun et al. 2022; Warner et al. 2013).

During the canning process, tuna meat is subjected to high heat and pressure (DeBeer et al. 2021), resulting in DNA degradation and complicating species identification (Mitchell and Hellberg 2016). Therefore, shorter fragments of DNA are typically targeted for identifying canned tuna species (Hellberg 2024). While DNA barcoding based on the cytochrome *c* subunit I (COI) gene is the regulatory standard for fish species identification (Handy et al. 2011), this method is not optimal for identifying closely related tuna species (Hellberg 2024; Klapper et al. 2023). Instead, alternate genetic makers, such as the mitochondrial control region (CR), have been established for tuna species (Cawthorn et al. 2011; Gordoa et al. 2017; Mitchell and Hellberg 2016; Roungchun et al. 2022; Viñas and Tudela 2009). A CR mini‐barcoding system utilizing a short (∼236 bp) region was developed by Mitchell and Hellberg (2016) to enable the identification of tuna species in heavily processed products. While this approach is promising, it has faced challenges with canned tuna products due to factors such as DNA fragmentation, limited primer annealing, and species mixtures (Chang et al. 2021; Emmi et al. 2023; Mitchell and Hellberg 2016; Roungchun et al. 2022). Furthermore, DNA barcoding is generally costly and time‐consuming due to the need for DNA sequencing (Chuang et al. 2012; Hellberg 2024; Klapper et al. 2023).

Targeted molecular methods, such as multiplex and real‐time PCR, offer rapid identification of species in mixtures, thereby overcoming some of the challenges of DNA barcoding (Chuang et al. 2012; Isaacs and Hellberg 2020; Krčmář et al. 2019; Lee et al. 2022; Lopez and Pardo 2005; Singh et al. 2024). Real‐time PCR utilizes fluorescence combined with species‐specific primers and probes to identify species (Krčmář et al. 2019; Lopez and Pardo 2005), while multiplex PCR utilizes a combination of species‐specific primers to identify multiple species in a single assay simultaneously (Lee et al. 2022). Although these targeted methods do not provide the same level of genetic information obtained with DNA barcoding, they have the potential to be implemented on‐site in real‐time, thus allowing species identification in a timely and cost‐effective manner (Isaacs and Hellberg 2020; Naaum et al. 2019). Therefore, this study aimed to compare CR mini‐barcoding to targeted (i.e., real‐time or multiplex) PCR‐based methods to determine the most effective approach for canned tuna species identification.

## 2 Materials and Methods

### 2.1 Sample Collection

Five species of raw tuna were collected as positive controls: albacore tuna, skipjack tuna, yellowfin tuna, bigeye tuna, and bluefin tuna (*Thunnus thynnus*). The raw skipjack tuna reference samples were donated by a canned tuna manufacturer in the United States. The remaining reference samples were purchased online and from local retailers (Orange, CA, USA). Raw tissue samples underwent species confirmation with DNA barcoding methods described previously (Kitch et al. 2023). A total of 24 canned tuna products were collected, representing four categories of canned tuna: albacore tuna (*n* = 6), skipjack tuna (*n* = 6), yellowfin tuna (*n* = 6), and light tuna (*n* = 6). The canned samples were purchased from grocery stores in Orange County, CA, USA. Additional product details can be found in the Supporting Information (Table S1). After collection, the entire contents of each canned sample were transferred to a 24‐oz Whirl‐Pak bag (Nasco, Fort Atkinson, WI, USA) and hand‐mixed for 60 s to homogenize the contents (Shokralla et al. 2015). Tissue samples (20–30 mg) were placed in sterile 1.5‐mL microcentrifuge tubes using sterile forceps and stored at −80°C until DNA extraction. All tissue samples were prepared in duplicate.

### 2.2 DNA Extraction

DNA extraction was performed on each duplicate tissue sample using the DNeasy Blood and Tissue Kit (Qiagen, Hilden, Germany), as described in Emmi et al. (2023). Each set of DNA extractions contained a negative control without tissue (i.e., reagent blank). Lysis was performed in a ThermoMixer C (Eppendorf, Hamburg, Germany) at 56°C and 300 rpm for 20 h. After extraction, DNA concentration and purity were measured using a NanoDrop One^c^ UV‐Vis Spectrophotometer (Thermo Fisher Scientific, Waltham, MA, USA). DNA extracts were stored at −80°C until further testing. The reagent blank from each DNA extraction batch was included as a negative control in CR mini‐barcoding, multiplex PCR, and real‐time PCR.

### 2.3 CR Mini‐Barcoding

All DNA extracts underwent CR mini‐barcoding using the reaction conditions described in Emmi et al. (2023). The reaction mixture contained 17.5 µL of Qiagen HotStar Taq Plus Master Mix (2×), 0.20 µM CR mini‐barcoding primers (Table 1), 4.20 µL of DNA template, and sterile water to bring the reaction volume to 35 µL. The primers were synthesized by Integrated DNA Technologies (Coralville, IA, USA). A nontemplate control, containing sterile water instead of DNA template, and a positive control reference sample were included in each PCR run. Cycling was carried out using a Mastercycler Nexus Gradient Thermal Cycler (Eppendorf) under the following conditions: 94°C for 15 min; 35 cycles of 94°C for 30 s, 49°C for 40 s, and 72°C for 1 min; and a final extension of 72°C for 10 min (Emmi et al. 2023). PCR products were confirmed with a 2.0% agarose E‐Gel EX (Invitrogen, Carlsbad, CA, USA) run on an E‐Gel Simple Runner (Invitrogen) for 30 min. The E‐Gels included an E‐Gel 1 Kb Plus DNA Ladder (Invitrogen) and were visualized with an E‐Gel Imager (Thermo Fisher Scientific).

**TABLE 1:** Primers and probes used in this study for CR mini‐barcoding, multiplex PCR, and real‐time PCR.

Assay	Target species	Primer/probe name	Primer direction	Sequence (5’ to 3’)	Concentration (µM)	Genetic marker length (bp)	Target gene^a^	Reference
CR mini‐barcoding	Tuna (universal primers)	Tuna CR_F2	Forward	CACGACGTTGTAAAACGACGCAYGTACATATATGTAAYTACACC	0.20	∼236	CR	Mitchell and Hellberg 2016
		Tuna CR_R1	Reverse	GGATAACAATTTCACACAGGCTGGTTGGTRGKCTCTTACTRCA	0.20			
		Tuna CR_R2	Reverse	GGATAACAATTTCACACAGGCTGGATGGTAGGYTCTTACTGCG	0.20			
Multiplex PCR	Bigeye	Obe‐F	Forward	ACTTGCATTCCCCCTATG	1.4	270	ATP6	Lee et al. 2022
		Obe‐R	Reverse	GCTGTTAGGATTGCCACAG				
	Skipjack	Kat‐F	Forward	GGTCCTAGCTCTTCTTGCA	1.2	238	Cytb	
		Kat‐R	Reverse	TGCAAGTGGGAAGAAGATG				
	Bluefin	Thy‐F	Forward	AACTCTTTATCGGGTGGGAG	0.8^b^	200	NADH5	
		Thy‐R	Reverse	AGCGGTTACGAACATTTGCTTTC				
	Albacore	Ala‐F	Forward	GTTTCGTGATCCTGCTAGTG	1.2^b^	178	Cytb	
		Ala‐R	Reverse	CCTCCTAGTTTGTTGGAATAGAT				
	Yellowfin	Alba‐F	Forward	CATGATTGCCCACGGACTTA	1.2	127	NADH4	
		Alba‐R	Reverse	TGTTGTTATAAGGGGCAGC				
Real‐time PCR	Albacore	Alb_Pardo_F	Forward	GCCTCTTTCTTCTTTATCTGCATCTAC	0.30	<100^c^	Cytb	Lopez and Pardo 2005
		Alb_Pardo_R	Reverse	TACTCCGATGTTTCATGTTTCTTTG	0.30			
		Alb_Pardo_P	Probe	(FAM)‐TCCACATCGCCGAGGCCTTTACTA‐(TAMRA)	0.25			
	Yellowfin	Yft_Pardo_F	Forward	CGAGGCCTTTACTACGGCTCTT	0.90	<100^c^	Cytb	
		Yft_Pardo_R	Reverse	CGGTCATCATAACTAGGAGTAGGAGTAC	0.90			
		Yft_Pardo_P	Probe	(FAM)‐CCTATACAAGGAAACATGAAA‐(MGB)	0.25			
	Skipjack	Skp_Krcmar_F	Forward	CTAGTGATTTGACACCTCGCT	0.5	85	NADH1	Krčmář et al. 2019
		Skp_Krcmar_R	Reverse	TTAGGCTTCAGGCACGACT	0.5			
		Skp_Krcmar_P	Probe	(FAM)‐ATCGCATTTGCAGGTCTACCCCCTCA‐(5IABkFQ)	0.1			

^a^CR = mitochondrial control region; ATP6 = ATP synthase membrane subunit 6; Cytb = cytochrome *b*; NADH5 = NADH dehydrogenase subunit 5; NADH4 = NADH dehydrogenase subunit 4; NADH1 = NADH dehydrogenase subunit 1.

^b^Primer concentration was increased twofold compared to that described in Lee et al. (2022) based on preliminary testing with reference samples.

^c^Exact length was not provided.

All PCR products confirmed with gel electrophoresis were treated with ExoSAP‐IT (Applied Biosystems, Santa Clara, CA, USA) according to the manufacturer's instructions. Bidirectional sequencing was carried out at Eurofins Genomics (Louisville, KY, USA) utilizing a BigDye Terminator v3.1 Cycle Sequencing Kit (Applied Biosystems) and a 3730xl DNA Analyzer (Applied Biosystems). Raw sequence data were assembled and edited with Geneious R7 (Biomatters, Ltd., Auckland, New Zealand) using the following quality control parameters: consensus sequences must be ≥180 bp in length and have <2% ambiguities (Emmi et al. 2023). The resulting sequences were queried in GenBank (National Center for Biotechnology Information [NCBI], Bethesda, MD, USA) using the basic local alignment search tool (BLAST) with the MegaBlast algorithm. The top species match was determined based on *E* value and percent identity. CR mini‐barcoding was considered successful if at least one of the duplicate DNA extracts from a given product passed quality control and resulted in species identification. All DNA sequences, quality control parameters, and top species matches can be found in the Supporting Information (Table S2).

### 2.4 Multiplex PCR

A multiplex PCR assay targeting bigeye, skipjack, bluefin, albacore, and yellowfin tuna was performed using primers described in Lee et al. (2022). The albacore and bluefin primer concentrations were increased twofold in the primer cocktail compared to the original conditions (Lee et al. 2022) based on preliminary testing with the raw tuna reference samples. The reaction mixture contained 12.5 µL of Qiagen HotStar Taq Plus Master Mix (2×), 5.00 µL of primer cocktail (Integrated DNA Technologies; Table 1), 2.00 µL of DNA template, and 5.50 µL of sterile water for a total reaction volume of 25.0 µL. Amplification was conducted using a Mastercycler Nexus Gradient Thermal Cycler under the following conditions: 95°C for 5 min; 40 cycles of 95°C for 30 s, 62°C for 30 s, and 72°C for 30 s; and a final extension of 72°C for 5 min (Lee et al. 2022). Each PCR batch included a nontemplate negative control and a positive control reference sample. PCR products were run for 30 min on a 4.0% agarose E‐Gel EX containing an E‐Gel 1 Kb Plus DNA Ladder. E‐Gels were run using an E‐Gel Simple Runner and visualized with an E‐Gel Imager. Multiplex PCR was considered successful if at least one of the duplicate DNA extracts from a given product resulted in species identification.

### 2.5 Real‐Time PCR

Real‐time PCR assays were performed targeting the top three species used in canned tuna products: albacore, skipjack, and yellowfin (NFI 2024b). Fewer species were targeted in real‐time PCR as compared to multiplex PCR because each species required a separate real‐time PCR assay. Real‐time PCR assays targeting the cytochrome *b* gene in albacore and yellowfin tuna were carried out using the primers and probes described in Lopez and Pardo (2005) with the following cycling conditions: 95°C for 10 min, followed by 40 cycles of 95°C for 15 s and 60°C for 1 min. Each reaction mixture contained 12.5 µL of TaqMan Universal Master Mix II (2×, Applied Biosystems), 0.3–0.9 µM PCR primers (Integrated DNA Technologies; Table 1), 0.25 µM probe (Thermo Fisher Scientific; Table 1), 2.00 µL of DNA template, and sterile water to bring the total reaction volume to 25 µL. A real‐time PCR assay targeting the NADH dehydrogenase subunit 1 gene in skipjack was carried out using the primers and probes described in Krčmář et al. (2019). The cycling conditions were as follows: 95°C for 15 min, followed by 40 cycles of 95°C for 15 s and 60°C for 1 min. Each reaction mixture contained 10.0 µL of TaqMan Universal Master Mix II (2×), 0.5 µM PCR primers (Integrated DNA Technologies; Table 1), 0.1 µM probe (Integrated DNA Technologies; Table 1), 4.00 µL of DNA template, and sterile water to bring the total reaction volume to 20 µL.

All real‐time PCR assays were run on a Rotor‐Gene Q (Qiagen). Each PCR batch included a nontemplate negative control and three positive controls of reference sample DNA from the target species (undiluted, 1:10 dilution, and 1:100 dilution). The results for a given sample were considered positive if a cycle threshold (Ct) value was recorded for the target signal and the positive controls but not for the negative controls. Due to nonspecific amplification observed in the negative controls late in the reaction for both the albacore and skipjack assays, a Ct cutoff of <32.0 was applied to the results of these assays to avoid false positives (Krčmář et al. 2019). To ensure the absence of cross‐reactivity with nontarget species, the reference samples of albacore, skipjack, yellowfin, bigeye, and bluefin tuna described in Section 2.1 were run against each real‐time PCR assay. Real‐time PCR was considered successful if at least one of the duplicate DNA extracts from a given product resulted in species identification.

### 2.6 Targeted Detection in Binary Species Mixtures

The top‐performing targeted PCR assay (i.e., real‐time PCR) was further tested for its ability to detect the target species at low levels in samples containing a mixture of two species. Binary species mixtures were prepared using DNA from raw tuna reference samples mixed at the following ratios based on DNA concentration: (1) 90% of the nontarget species:10% of the target species, (2) 95% of the nontarget species:5% of the target species, (3) 99% of the nontarget species:1% of the target species, and (4) 99.9% of the nontarget species:0.1% of the target species. The species mixtures with skipjack or yellowfin as the target species contained albacore as the nontarget species, and the species mixtures with albacore as the target species contained yellowfin as the nontarget species. The mixtures were prepared in Tris‐EDTA (TE) buffer solution using a DNA concentration of 25 ng/µL. The binary species mixtures were tested with and without heat treatment at 95°C for 30 min to simulate canning conditions (Krčmář et al. 2019). All fresh and heat‐treated (“simulated canned”) binary species mixtures underwent real‐time PCR in triplicate using the conditions described in Section 2.5.

## 3 Results and Discussion

### 3.1 CR Mini‐Barcoding

**FIGURE 1:**
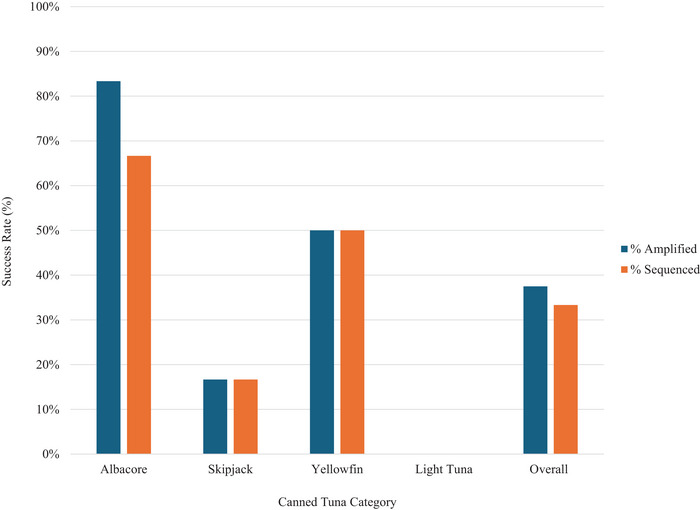
CR mini‐barcoding amplification and sequencing success rates for canned tuna samples separated by product category (*n* = 6 per category). Each sample was tested with duplicate DNA extracts, and success was based on a positive result for at least one DNA extract.

The results of CR mini‐barcoding enabled tuna species identification in eight of the 24 canned samples for an overall sequencing success rate of 33% (Figure 1). Specifically, three samples were identified as albacore, three as yellowfin tuna, one as bluefin tuna, and one as bigeye tuna (Table 2). Among the samples identified, the top species matches in GenBank showed 87%–100% genetic similarity to the query sequence (Table 2). Amplification and sequencing success varied depending on the canned tuna product category (Figure 1). The greatest sequencing success was observed among canned tuna samples labeled as albacore (66.7%) and yellowfin (50%). On the other hand, canned products labeled as skipjack and light tuna showed the lowest amplification/sequencing success rates, at 17% and 0%, respectively (Figure 1).

**TABLE 2:** Species detected in canned tuna samples following analysis with CR mini‐barcoding, multiplex PCR, and real‐time PCR. All samples were tested in duplicate. A dash indicates that no identification was made.

Sample	Declared species	CR mini‐barcoding (% identity)^a^	Multiplex PCR detection	Real‐time PCR detection (Ct value)^b^
F01	Light tuna	—	—	Skipjack (25.3)
F02	Light tuna	—	—	Skipjack (21.6)
F03	Light tuna	—	—	Skipjack (21.9), yellowfin (34.1)
F04	Light tuna	—	—	Skipjack (25.3)
F05	Light tuna	—	—	Skipjack (24.5), yellowfin (33.1)
F06	Light tuna	—	—	Skipjack (23.3)
F07	Albacore	Albacore (87.1%)	—	Albacore (22.4)
F08	Albacore	Albacore (99.0%)	Albacore	Albacore (22.4)
F09	Albacore	—	—	Albacore (23.4)
F10	Albacore	—	—	Albacore (23.4)
F11	Albacore	Albacore (96.2%)	—	Albacore (23.4)
F12	Albacore	Bluefin^c^ (99.6%)	—	Albacore (24.1)
F13	Skipjack	—	—	Skipjack (23.5)
F14	Skipjack	—	—	Skipjack (22.7)
F15	Skipjack	—	Yellowfin	Skipjack (19.4), yellowfin (31.8)
F16	Skipjack	Bigeye (94.9%)	Skipjack	Skipjack (24.1), yellowfin (25.2)
F17	Skipjack	—	—	Skipjack (23.1)
F18	Skipjack	—	—	Skipjack (22.5)
F19	Yellowfin	Yellowfin (99.6%)	Yellowfin	Yellowfin (23.7), albacore (31.2)
F20	Yellowfin	—	—	Yellowfin (29.5)
F21	Yellowfin	Yellowfin (91.8%)	Yellowfin	Yellowfin (24.0), albacore (28.7)
F22	Yellowfin	Yellowfin (100%)	Yellowfin	Yellowfin (23.9)
F23	Yellowfin	—	Yellowfin	Yellowfin (24.3)
F24	Yellowfin	—	—	Yellowfin (26.8)

^a^Top genetic match to sequences in GenBank using the basic local alignment search tool (BLAST). In cases where the % identity obtained with each duplicate sample was different, the average is reported.

^b^In cases where the Ct value obtained with each duplicate sample was different, the average is reported.

^c^Result should be interpreted with caution due to the possibility of species introgression between albacore and bluefin tuna.

The overall species identification rate determined in the current study (33%) is slightly lower compared to the identification rates (42%–50%) reported in previous studies that have applied this method to canned tuna (Emmi et al. 2023; Mitchell and Hellberg 2016; Roungchun et al. 2022). The differences in identification rates are likely due to variations in sampling design, as factors such as packing medium and tuna species can influence the success rate (Aksun Tumerkan 2022; Chapela et al. 2007; Emmi et al. 2023; Mitchell and Hellberg 2016). The generally low success rates obtained with CR mini‐barcoding may be attributed to DNA degradation during canning (Kappel et al. 2017; Klapper et al. 2023; Shokralla et al. 2015). Prior research has reported difficulty amplifying 200‐ to 400‐bp fragments of canned tuna, suggesting that DNA may be degraded to <200 bp during canning (Lin and Hwang 2007; Quinteiro et al. 1998). Therefore, it is possible that the DNA in some of the canned tuna products tested in the current study was too fragmented to amplify across the 236‐bp CR mini‐barcode. The lack of success observed in the current study in identifying skipjack and light tuna is consistent with prior research that utilized this method (Emmi et al. 2023; Mitchell and Hellberg 2016; Roungchun et al. 2022). Given that light tuna sold in the United States is primarily composed of skipjack (NFI 2024b), the reduced success in these products is likely due to inadequate primer binding to skipjack combined with the possibility of species mixtures (Emmi et al. 2023; Mitchell and Hellberg 2016; Roungchun et al. 2022).

Of the eight products identified with CR mini‐barcoding, six confirmed the presence of the declared species (albacore or yellowfin tuna; Table 2). One product labeled as skipjack (F16) was identified as bigeye tuna with 94.9% genetic identity (Table 2). The detection of bigeye in products labeled as skipjack has been reported previously and may be due to accidental inclusion (Mariani et al. 2015; Roungchun et al. 2022; Sotelo et al. 2018). While the species are morphologically distinct, they inhabit the same waters, introducing the possibility of bycatch (NOAA Fisheries 2024; Roungchun et al. 2022). An additional product labeled as albacore (F12) was identified as bluefin tuna with 99.6% genetic identity (Table 2). However, this identification should be interpreted with caution due to the possibility of species introgression between albacore and bluefin tuna (Mitchell and Hellberg 2016; Sotelo et al. 2018; Viñas and Tudela 2009). Further testing with a secondary marker, such as the first internal transcribed spacer region (ITS1), would be required for species confirmation of this sample (Mitchell and Hellberg 2016).

### 3.2 Multiplex PCR

Multiplex PCR enabled tuna species identification in seven of the 24 canned tuna products for a 29% detection rate (Table 2). Specifically, multiplex PCR detected albacore tuna in one sample, yellowfin tuna in five samples, and skipjack tuna in one sample. The relatively low success rate observed for multiplex PCR may be due, in part, to the ≥200 bp amplicon length for three (bigeye, skipjack, and bluefin) of the five target species. As mentioned above, DNA may be degraded during processing to <200 bp (Lin and Hwang 2007; Quinteiro et al. 1998). Along these lines, the greatest identification success was observed for yellowfin tuna, which had the shortest amplicon length (127 bp).

The identification rate reported in the current study for multiplex PCR (29%) is low compared to previous work by Lee et al. (2022), which reported 100% identification success for 12 commercially canned tuna products. The reduced identification rate in the current study may be due to differences in sampling design and methodological details. While the primer sequences and cycling conditions were consistent across studies, the current study used a different DNA extraction method and a modified PCR mixture compared with that described in Lee et al. (2022). For example, preliminary testing with raw tuna reference samples showed a lack of amplification using the albacore and bluefin tuna primer concentrations reported by Lee et al. (2022), and they were therefore increased twofold to allow for the detection of these species. Further optimization of the multiplex PCR method in a multilaboratory study may improve its success rate.

Six of the seven products identified with multiplex PCR tested positive for the declared species (Table 2). One of these products was labeled as albacore tuna, one was labeled as skipjack tuna, and four were labeled as yellowfin tuna. The remaining product (F15) was labeled as skipjack tuna but identified as yellowfin tuna. Similarly, previous studies have reported the detection of yellowfin in processed products declared to be skipjack (Emmi et al. 2023; Lee et al. 2022; Mariani et al. 2015; Roungchun et al. 2022; Servusova and Piskata 2021; Sotelo et al. 2018).

### 3.3 Real‐Time PCR

Real‐time PCR achieved a 100% detection rate across all canned products, successfully identifying at least one tuna species in each sample (Table 2). These results are in agreement with the original studies that developed these assays, which reported high success in the detection of tuna species in commercially canned (Krčmář et al. 2019) or simulated canned (Lopez and Pardo 2005) samples. These assays targeted relatively short (<100 bp) regions of DNA (Table 1), which likely contributed to the success of these methods.

Overall, the results of real‐time PCR revealed the presence of a single species in 75% of the products, while two species were detected in the remaining 25% of products (Table 2). Notably, 100% of canned products labeled as albacore were confirmed to be albacore (*n* = 6), with no additional species detected. Albacore was also detected as a secondary species in two products labeled as yellowfin tuna (F19 and F21). The detection of albacore in canned yellowfin tuna has been reported previously (Mariani et al. 2015). Skipjack was detected in all canned products labeled as skipjack (*n* = 6) or light tuna (*n* = 6). Similarly, Krčmář et al. (2019) reported positive detection of skipjack in all 28 commercial cans labeled as such when using the same assay. Yellowfin was detected in 100% of canned products labeled as yellowfin and as a secondary species in 17% of products—specifically in cans labeled as light tuna (*n* = 2) and skipjack (*n* = 2). As mentioned above, yellowfin has been detected in skipjack products in several prior studies (Emmi et al. 2023; Lee et al. 2022; Mariani et al. 2015; Roungchun et al. 2022; Servusova and Piskata 2021; Sotelo et al. 2018). In most cases (*n* = 5) of secondary species detection, the Ct value for the secondary species was relatively high (28.7–34.1; Table 2), indicating that the species was present at a relatively low level. The presence of a low level of secondary species may have been due to intentional substitution or unintentional inclusion during harvesting or processing (Roungchun et al. 2022).

#### 3.3.1 Binary Species Mixtures

Given the high success rate for real‐time PCR obtained in the current study, additional testing was carried out using binary species mixtures of positive control samples to assess the sensitivity of the method. As shown in Table 3, real‐time PCR consistently detected albacore and yellowfin tuna at levels as low as 0.1% in binary species mixtures. This was true for both fresh and simulated canned samples, with detection of the target species in 100% of samples. Skipjack tuna was consistently detected at a level of 1.0% in binary species mixtures, with detection in only one of three (33%) samples tested at the 0.1% level. These results were consistent for both the fresh and simulated canned samples of skipjack.

**TABLE 3:** Results of real‐time PCR testing of binary species mixtures containing 0.1%–10% of the target species mixed with a nontarget tuna species. The mixtures were tested without heat treatment (“fresh”; *n* = 3) and with heat treatment at 95°C for 30 min (“simulated canned”; *n* = 3).

Target species	Nontarget species	Proportion of target species in binary mixture	Target species detection in real‐time PCR	
			Fresh samples	Simulated canned samples
Skipjack tuna	Albacore tuna	0.1%	+ − −	+ − −
		1.0%	+ + +	+ + +
		5.0%	+ + +	+ + +
		10%	+ + +	+ + +
Yellowfin tuna	Albacore tuna	0.1%	+ + +	+ + +
		1.0%	+ + +	+ + +
		5.0%	+ + +	+ + +
		10%	+ + +	+ + +
Albacore tuna	Yellowfin tuna	0.1%	+ + +	+ + +
		1.0%	+ + +	+ + +
		5.0%	+ + +	+ + +
		10%	+ + +	+ + +

*Note*: “+” = target species detected; “−” = target species not detected.

Lopez and Pardo (2005) previously found that the real‐time PCR assay used in this study could reliably detect albacore and yellowfin species in binary mixtures containing as little as 10% of the target species. However, the authors did not report testing the assay against mixtures containing lower levels of the target species. The findings of the current study reveal heightened assay sensitivity, with consistent detection of the target species at levels as low as 0.1% in a binary species mixture. Regarding the skipjack real‐time PCR assay, Krčmář et al. (2019) reported a detection limit of 0.0032 ng/µL for skipjack in heat‐treated single‐species samples (the authors did not report testing of binary species mixtures). In comparison, the current study demonstrated the detection of skipjack at concentrations as low as 0.005 ng/µL in binary mixtures (0.1 ng of target species DNA in a 20‐µL reaction), although consistent detection was not achieved at this concentration. These findings suggest that the real‐time PCR assays utilized in the current study are highly sensitive, even when tested against heat‐treated samples, with detection in binary species mixtures as low as 0.1%–1.0%.

### 3.4 Comparison of PCR‐Based Methods

When comparing all three PCR‐based methods, the highest identification rate was found with real‐time PCR (100%), followed by CR mini‐barcoding (33%) and multiplex PCR (29%). In addition to real‐time PCR being a highly sensitive assay, its success may also have been due to the short (<100 bp) DNA fragments targeted (Table 1). In comparison, multiplex PCR and CR mini‐barcoding targeted slightly longer fragments of 127–270 and ∼236 bp, respectively. Regarding species identification, CR mini‐barcoding and multiplex PCR confirmed the presence of albacore or yellowfin tuna in several samples; however, both methods struggled with the identification of skipjack tuna (Table 2). On the other hand, real‐time PCR consistently identified the declared species (i.e., skipjack, albacore, or yellowfin tuna) in the products. Furthermore, real‐time PCR enabled the identification of multiple species within a single product. For example, CR mini‐barcoding and multiplex PCR detected yellowfin (the declared species) in samples F19 and F21, whereas real‐time PCR detected the presence of yellowfin and albacore (Table 2). The Ct values obtained for albacore in these cases were relatively high, indicating a low level of this species in the products.

While real‐time PCR showed the greatest success, it can only reveal the presence or absence of the targeted species. On the other hand, CR mini‐barcoding allows for the identification of a broader range of species in products, which can provide a comprehensive picture of the species content in a given product. For example, real‐time PCR detected the presence of skipjack and yellowfin in a product declared to be skipjack (F16), while CR mini‐barcoding detected bigeye (Table 2). These results suggest the presence of multiple species in a single product. Indeed, small bigeye and yellowfin tuna are often packed together in cans due to the similar appearance of their meat (DeBeer et al. 2021). To expand the range of species identified by real‐time PCR, future research should consider incorporating an assay targeting bigeye tuna (e.g., Chuang et al. 2012). In other instances, the identifications obtained with real‐time PCR were confirmed with CR mini‐barcoding, which provides additional genetic information through DNA sequences. For example, three products labeled as albacore were determined to contain albacore with real‐time PCR and confirmed as albacore by CR mini‐barcoding, with genetic matches of 87%–99% to sequences in GenBank (Table 2). The use of multiple DNA analytical techniques and multiple genetic loci for species identification was previously suggested by Singh et al. (2024) to ensure increased accuracy and confidence in the results. Therefore, the combined use of real‐time PCR and CR mini‐barcoding is recommended for the identification of canned tuna species.

A comparison of the three methods based on ease of use, cost, and time can be found in Table 4, with additional pricing details included in the Supporting Information (Table S3). It should be noted that these parameters were determined based on the methods reported in this study and may vary depending on the supplies, manufacturers, and third‐party sequencing laboratories utilized. Overall, multiplex PCR and real‐time PCR were found to be the most cost‐effective methods, with a price per sample of US$6 when testing for all species targeted with these assays in the current study. In comparison, the price per sample for DNA barcoding was US$18 due to the additional expenses associated with DNA sequencing. When the costs were calculated based on batches of 10 samples, multiplex PCR became the most cost‐effective method (US$43 per batch) compared to US$53 for real‐time PCR and US$160 for CR mini‐barcoding. Real‐time PCR was the most user‐friendly method, with only a short (0.5 h per target species) PCR preparation step needed, followed by a PCR run and results interpretation. On the other hand, multiplex PCR was slightly more complex (0.75 h technician time) due to the need for gel electrophoresis following PCR. However, real‐time PCR required a separate assay for each species targeted, making it more time‐consuming than multiplex PCR when testing for multiple species. Due to the numerous steps involved, CR mini‐barcoding required more technician time (2 h) than the targeted methods. When comparing the total time needed for all three methods, multiplex PCR required the shortest amount of time (3 h), followed by real‐time PCR (6 h for all three species) and CR mini‐barcoding (2 days).

**TABLE 4:** Comparison of ease of use, cost, and time associated with each method compared in this study.

Method	Relative ease of use	Price per sample (USD)^a^	Price per 10 samples (USD)^a^	Hands‐on technician time per 10 samples	Total time required to analyze 10 samples	Comments
CR mini‐barcoding	Moderate–difficult	$18	$160	2 h^b^	2 days	Universal assay
Real‐time PCR	Easy–moderate	$1–2 per target species; $6 for all three species	$14–22 per target species; $57 for all three species	0.5 h per target species; 1.5 h for all three species	2 h per target species; 6 h for all three species	Separate assays must be run for each target species
Multiplex PCR	Moderate–difficult	$6	$43	0.75 h	3 h	Assay simultaneously tests for five species

^a^Prices are rounded to the nearest dollar amount and are based on the 2024 list prices from the manufacturers’ websites. Prices do not include the cost of DNA extraction, the positive and negative controls, or instrumentation. The price per 10 samples is proportionally less than the price per sample for CR mini‐barcoding and multiplex PCR due to the use of a single molecular weight ladder per batch of 10 samples. The price per 10 samples for real‐time PCR is proportionally different than the price per sample due to rounding.

^b^Does not include technician time at the sequencing facility.

## 4 Conclusion

This study revealed the effectiveness of real‐time PCR for species identification in canned tuna. In addition to successfully identifying all 24 canned tuna products tested, real‐time PCR was also the most user‐friendly method. Additionally, testing of binary species mixtures showed that real‐time PCR was highly sensitive, with detection of the target species at levels of 0.1%–1% in both fresh and simulated canned samples. While both real‐time PCR and multiplex PCR were found to be time‐ and cost‐effective, multiplex PCR was unable to identify species in the majority of products. CR mini‐barcoding could only identify species in one third of products, and it required additional time and expense compared to the targeted approaches; however, CR mini‐barcoding provides a greater level of genetic information. Therefore, it is recommended that real‐time PCR is used as a rapid screening method in concert with CR mini‐barcoding to provide sequencing‐based species confirmation. Further optimization of CR mini‐barcoding should be conducted to improve sequencing‐based species identification rates in commercially canned tuna. The use of alternative genetic markers and/or sequencing approaches should also be considered.

## Author Contributions

**Chloe P. Castanon**: conceptualization, methodology, validation, formal analysis, investigation, data curation, writing – original draft, visualization, supervision, funding acquisition. **Denise Hernandez**: methodology, validation, formal analysis, investigation, data curation, writing – original draft, visualization. **Akshay N. Khetrapal**: conceptualization, methodology, validation, formal analysis, investigation, data curation, writing – original draft, visualization, funding acquisition. **Rosalee S. Hellberg**: conceptualization, methodology, formal analysis, resources, data curation, writing – original draft, supervision, project administration, funding acquisition.

## Conflicts of Interest

The authors declare no conflicts of interest.

## Supporting information




**Supporting Table S1**: provides product details for canned tuna samples.
**Supporting Table S2**: provides sequencing data for canned tuna samples.
**Supporting Table S3**: provides detailed pricing information for each assay.
