# Supplementary material for: Comparison of PCR‐Based Methods for the Detection of Canned Tuna Species
Source: J Food Sci. 2025 Sep 19;90(9):e70424. doi: 10.1111/1750-3841.70424 (PMC12447540; doi:10.1111/1750-3841.70424)
Supplement: Supplementary file 1 — Supporting Table S1: provides product details for canned tuna samples. Supporting Table S2: provides sequencing data for canned tuna samples. Supporting Table S3: provides detailed pricing information for each assay. [file JFDS-90-0-s001.docx]

**Table S1.** Product details for canned tuna samples tested in this study.

| **Sample Number** | **Declared Species on Label** | **Ingredients List (exact wording)** | **Price/unit (USD)** |
| --- | --- | --- | --- |
| F01 | Light tuna | light tuna, water, vegetable broth, sea salt | 1.29 |
| F02 | Light tuna | light tuna, vegetable broth, salt | 1.59 |
| F03 | Light tuna | chunk light tuna, water, vegetable broth (soy), salt | 1.29 |
| F04 | Light tuna | chunk light tuna, soybean oil, vegetable broth, salt | 3.49 |
| F05 | Light tuna | light tuna, soybean oil, water, vegetable broth, salt | 1.00 |
| F06 | Light tuna | light tuna, water, vegetable broth flavor, salt | 0.88 |
| F07 | Albacore tuna | chunk white albacore tuna, extra virgin olive oil, water, sea salt | 3.99 |
| F08 | Albacore tuna | albacore tuna chunks, olive oil, water, salt | 5.69 |
| F09 | Albacore tuna | albacore tuna (Thunnus alalunga) and sea salt | 3.99 |
| F10 | Albacore tuna | white tuna, water, vegetable broth, salt, pyrophosphate | 1.99 |
| F11 | Albacore tuna | white tuna, water, vegetable broth (pea, carrot), salt, pyrophosphate added | 1.89 |
| F12 | Albacore tuna | solid white tuna, olive oil, salt | 2.29 |
| F13 | Skipjack tuna | tuna, water, sea salt | 1.99 |
| F14 | Skipjack tuna | skipjack tuna, sea salt | 4.99 |
| F15 | Skipjack tuna | skipjack tuna chunks, water, salt | 3.49 |
| F16 | Skipjack tuna | skipjack tuna, canola oil, water, vegetable broth (onion, carrot, cabbage) | 2.99 |
| F17 | Skipjack tuna | skipjack tuna, spring water | 1.99 |
| F18 | Skipjack tuna | skipjack tuna, spring water, sea salt | 1.99 |
| F19 | Yellowfin tuna | tuna fish, water, sea salt | 3.99 |
| F20 | Yellowfin tuna | solid light yellowfin tuna, olive oil, sea salt | 3.79 |
| F21 | Yellowfin tuna | yellowfin tuna, extra virgin olive oil, sea salt | 2.50 |
| F22 | Yellowfin tuna | yellowfin tuna, olive oil, water, vegetable broth | 3.99 |
| F23 | Yellowfin tuna | yellowfin tuna, sea salt | 2.79 |
| F24 | Yellowfin tuna | solid light tuna, olive oil, salt | 2.29 |

**Table S2.** CR mini-barcoding data for canned tuna samples identified through DNA sequencing in this study. All samples were tested in duplicate (indicated by -1 and -2). Samples are only shown here if they produced sequences that passed quality control.

| **Sample Number** | **DNA Sequence** | **Sequence Length** | **% Ambi-**  **guities** | **Sequence Quality (%HQ)** | **Top Species Match in BLAST** | **Common Name^a^** | **Query Coverage (%)** | **E Value** | **% Identity** |
| --- | --- | --- | --- | --- | --- | --- | --- | --- | --- |
| F07-1 | TATATGTATTAAAACCATAACTAGTATTTAACCATTCATNTGTNAACATATCATGAAGGCTTGCATAAGACATATCAACCATTCCCCAACACTCTGGTTATATCCGGGAGATAAACGAAATTTAAGACCTAACATAAACCTAAATCGTCTAARCCCTACCCAGGCCCCCCATTCTTAAAAATTTCGAAAATTATG | 195 | 1.54 | 28.70 | Thunnus alalunga | Albacore tuna | 98 | 1.00E-53 | 87.11 |
| F08-1 | ATAACTATATTTAAAACATATATAATAATGCTTTAGGACATATATGTATTAAAACCATAACTAGTATTTAACCATTCATATGTCAACATATCATGAAGGTTTGCATAAGACATATCAATCACCTCTCAACACTCTAGTTATAACTGGATGATTAAACGAAATTTAAAACCTAACTTAAACCTAAACCGTCTAAGCCACACCAAGTCCCCCCATCTCTGAAATCTCGTAAATTCATA | 236 | 0.00 | 55.10 | Thunnus alalunga | Albacore tuna | 100 | 1.00E-109 | 97.88 |
| F08-2 | ATAACTATATTTAAAACATATATAATAATGCTTTAGGACATATATGTATTAAAACCATAACTAGTATTTAACCATTCATATGTCAACATATCATGAAGGTTTGCATAAGACATATCAATCACCTCTCAACACTCTAGTTATAACTGTATGATTAAACGAGATTTAAGACCTAACATAAACCTAAATCGTCTAAGCCACACCAAGTCCCCCCATCTCTGAAATCTCGTAAATTCATA | 236 | 0.00 | 99.60 | Thunnus alalunga | Albacore tuna | 100 | 5.00E-118 | 100 |
| F11-2 | ATAACTATATTTGAAACATATATAATAAAGCTTTAGGTCATATACGTATTAAACCCATACCTAGTATTTAACCATTCATATGTCAACATATCACGAAAACTTACATAAARCATAACAATCMTCCCCCAACACTCTACTTATATCACGTAATTAAACGAGATTTAAGACCTAACACAAACCTAAATCGTCTAAGCCACACCAAGTCCCCCCATCTCTAAAATCTGGTAAACTTATG | 235 | 0.85 | 49.80 | Thunnus alalunga | Albacore tuna | 100 | 1.00E-103 | 96.17 |
| F12-1 | ATAACTATATTTGAAACATATATAATAATGCTTTAGGACATACATGTATTAAAACCATAACTAGTATTTAACCATTCATATGTCAACATATCATGAAGACTTACATAAAACATAACAACCATCTCCCAACACTTTATTTATATCACGTAATTAGACGAGATTTAAGACCTAACATAAACCTAAATCGTCTAAGCCATACCAAGTCCCCCCATCTCTAAAATCTAGTAAACTTATG | 235 | 0.00 | 55.30 | Thunnus thynnus^b^ | Bluefin tuna^b^ | 100 | 8.00E-116 | 99.57 |
| F16-2 | TATATGTATTAGAAACCATTACTAGTACTAAACCATTNNATGTCAACAAACAATGAAGACTTACATAAACCATACAGNTATATTCCAATATTCAAGTTAAGTCGAGTAATTAAACGAGATTTAAGACCTACCACAAACACTAAATCGTCTAAGCCATACCAAGTCTCCTCATCCCTGAAATGAGTGAAATATTA | 194 | 0.52 | 55.20 | Thunnus obesus | Bigeye tuna | 100 | 3.00E-79 | 94.87 |
| F19-1 | ATACTCATATATCGACCATATATAATAATGCTTTAGGACATATATGTATTAAAACCATTACTAGTACTAAACCATTCATATGTCAACAAACAATGAAGACTTACATAAACCATACAGATATATCTTAGTATTCAACCTAAGTCAAGTAATTAAACGAGATTTAAGACCTACCATAACAACTAAATCGTCTAAGCCATACCAAGTATCCCCATTCCTGAAATCGGGTAAATTTAAG | 235 | 0.00 | 93.20 | Thunnus albacares | Yellowfin tuna | 100 | 8.00E-116 | 99.57 |
| F19-2 | ATACTCATATATCGACCATATATAATAATGCTTTAGGACATATATGTATTAAAACCATTACTAGTACTAAACCATTCATATGTCAACAAACAATGAAGACTTACATAAACCATACAGATATATCTTAGTATTCAACCTAAGTCAAGTAATTAAACGAGATTTAAGACCTACCATAACAACTAAATCGTCTAAGCCATACCAAGTATCCCCATTCCTGAAATCGGGTAAATTTAAG | 235 | 0.00 | 96.60 | Thunnus albacares | Yellowfin tuna | 100 | 8.00E-116 | 99.57 |
| F21-2 | CCATATAGAAAAATGCTATAGGACATATATGTATTAAAACCATCACTAGTGGTAANCCATTTATATGTCCACAAATAATGAAGATTTGCATCATCCAANNAGATATTTTTTAATATTCAATCTAAGTCAAGTAATTAAACGAGATTTAAGACCTACCATAACAACTAAATCGTCTAAGCCATACCAAGTATCCCCATTCCTAAAGTCAAGTAAATTTAAG | 220 | 1.36 | 22.30 | Thunnus albacares | Yellowfin tuna | 100 | 7.00E-81 | 91.82 |
| F22-1 | ATATTCATATATCGACCATATATAATAATGCTTTAGGACATATATGTATTAAAACCATTACTAGTACTAAACCATTCATATGTCAACAAACAATGAAGATTTACATAAACCATACAAATATATCTTAACATTCAACCTAAGTCAAGTAATTAGACGAGATTTAAGACCTACCATAATAACTAAATCGTCTAAGCCATACCAAGTATCCCCATTCCTAAAGTCAAGTAAATTTAAG | 235 | 0.00 | 85.50 | Thunnus albacares | Yellowfin tuna | 100 | 2.00E-117 | 100 |

^a^According to the FDA Seafood List

^b^Result should be interpreted with caution due to the possibility of species introgression between albacore and bluefin tuna.

**Table S3.** Detailed pricing information for each method described in this study. All prices are in USD.

| **Assay** | **Price per sample^a^** | | | | | | | | **Total cost per sample** |
| --- | --- | --- | --- | --- | --- | --- | --- | --- | --- |
|  | **Primers** | **Probes** | **Commercial master mix** | **E-gel (price per well)** | **DNA ladder** | **PCR clean up** | **PCR tubes** | **Third-party sequencing** |  |
| CR mini-barcoding | $0.39 | N/A | $1.18 | $2.12 | $2.37 | $1.47 | $0.11 | $10.50 | $18.14 |
| Real-time PCR (albacore) | $0.02 | $0.40 | $1.60 | N/A | N/A | N/A | $0.09 | N/A | $2.11 |
| Real-time PCR (yellowfin) | $0.06 | $0.46 | $1.60 | N/A | N/A | N/A | $0.09 | N/A | $2.21 |
| Real-time PCR (skipjack) | $0.02 | $0.03 | $1.28 | N/A | N/A | N/A | $0.09 | N/A | $1.42 |
| Multiplex PCR | $0.30 | N/A | $1.18 | $2.49 | $2.37 | N/A | $0.05 | N/A | $6.39 |

^a^Prices are based on the 2024 list prices from the manufacturers’ websites. All manufacturer names are provided within the main text of the manuscript. Prices do not include the cost of DNA extraction, the positive and negative controls, or instrumentation.
